# Pharmacokinetic Characterization of (Poly)phenolic Metabolites in Human Plasma and Urine after Acute and Short-Term Daily Consumption of Mango Pulp

**DOI:** 10.3390/molecules25235522

**Published:** 2020-11-25

**Authors:** Jiayi Fan, Di Xiao, Liyun Zhang, Indika Edirisinghe, Britt Burton-Freeman, Amandeep K. Sandhu

**Affiliations:** Department of Food Science and Nutrition, Center for Nutrition Research, Institute for Food Safety and Health, Illinois Institute of Technology, Chicago, IL 60616, USA; fjiayi@hawk.iit.edu (J.F.); dxiao6@iit.edu (D.X.); lzhan134@hawk.iit.edu (L.Z.); iedirisi@iit.edu (I.E.); bburton@iit.edu (B.B.-F.)

**Keywords:** mango, polyphenols, gallotannins, pharmacokinetics, plasma, urine

## Abstract

Pharmacokinetic (PK) evaluation of polyphenolic metabolites over 24 h was conducted in human subjects (*n* = 13, BMI = 22.7 ± 0.4 kg/m^2^) after acute mango pulp (MP), vitamin C (VC) or MP + VC test beverage intake and after 14 days of MP beverage intake. Plasma and urine samples were collected at different time intervals and analyzed using targeted and non-targeted mass spectrometry. The maximum concentrations (C_max_) of gallotannin metabolites were significantly increased (*p* < 0.05) after acute MP beverage intake compared to VC beverage alone. MP + VC beverage non-significantly enhanced the C_max_ of gallic acid metabolites compared to MP beverage alone. Pyrogallol (microbial-derived metabolite) derivatives increased (3.6%) after the 14 days of MP beverage intake compared to 24 h acute MP beverage intake (*p* < 0.05). These results indicate extensive absorption and breakdown of gallotannins to galloyl and other (poly)phenolic metabolites after MP consumption, suggesting modulation and/or acclimation of gut microbiota to daily MP intake.

## 1. Introduction

Mango (*Mangifera indica* L.) is one of the most economically important fruits in the world due to its appealing taste and high nutritional value [1]. Mango has a unique and diverse phytochemical profile [2] consisting of carotenoids and a number of polyphenol compounds including mangiferin, gallotannins, catechin, quercetin, kaempferol, gallic acid, ellagic acid and other phenolic acids [3]. The polyphenolic profile of mango is associated with reducing the risk of developing a number of chronic diseases and their related complications [4,5]. Several in vitro and in vivo animal studies support anti-diabetic [6,7], anti-cancer [8,9], anti-inflammatory [10,11], anti-oxidant [12,13] and anti-bacterial [14] activities linked with the intake of mango pulp, peel, seed, juice, extracts and other mango products. Additionally, bioavailability and bioaccessibility analyses of isolated compounds (mangiferin) or extracts from mango leaf and mango seed kernel have been conducted in pre-clinical and in vitro models [15,16,17,18,19]. However, mango pulp (MP) consumed by humans involves delivery/absorption of bioactive components from a complex food matrix potentially influencing bioavailability. Only a few studies have assessed the absorption and metabolism of polyphenols from mango fruit in humans [4,20,21].

A previous study identified and quantified five compounds in plasma and seven compounds in urine after “Ataulfo” mango consumption (500 g of flesh or 721 g of juice) [21]. Barnes et al. reported seven galloyl metabolites in urine after a 10-day consumption of 400 g of MP from the Keitt variety [20]. The same research group reported five galloyl-derivatives in plasma after 6 weeks of mango supplementation [4]. Collectively, only a few metabolites have been reported in plasma after MP intake and mangiferin has never been detected in previous studies.

Therefore, the first objective of this study was to characterize and investigate the absorption/kinetic profile of mango (poly)phenols and their metabolites in plasma over 24 h after MP consumption.

Black pepper (piperine), vitamin C (VC) and vitamin E have all been documented to enhance the absorption of polyphenol compounds when added to foods and beverages [22,23]. Vitamin C enhanced the absorption of green tea polyphenols in humans [24] and worked synergistically with oat (poly)phenolics and almond skin flavonoids to protect low density lipoprotein (LDL) against oxidation in a hamster and human model [25,26]. Vitamin C is inherent to the mango fruit; however, amounts may not be sufficient to stabilize polyphenols during digestion and transit through the gastrointestinal tract to enhance absorption in the small intestine or help maintain structural integrity for microbial metabolism in the large intestine. So, the second objective of this study was to test the hypothesis that concomitant intake of VC with MP would enhance absorption of mango (poly)phenols by increasing the number and/or concentration of mango (poly)phenolic metabolites.

Our previous work with berries indicates that regular intake or exposure to fruit polyphenols increases concentration of some metabolites in blood [27,28,29,30]. Likewise, increased excretion of certain mango metabolites was observed in urine after a 10-day intake of MP, of which some were microbial-derived [20]. Therefore, the third objective of this study was to assess the influence of daily intake of MP for 14 days on (poly)phenolic metabolite profiles.

## 2. Results and Discussion

### 2.1. Mango Pulp

Among the 46 tentatively identified compounds in MP (Appendix A, Table A1), gallotannins accounted for a large proportion of total MP polyphenols, including galloyl diglucoside, galloyl glucose isomers, galloyl-quinic acid, trigalloyl glucose isomers, tetragalloyl glucose isomers, and pentagalloyl glucose. Besides gallic acid derivatives, mangiferin, the signature compound of mango [31], was also identified along with quercetin, kaempferol, benzoic acids, protocatechuic acids, catechin, coumarins and ellagic acid compounds. These compounds have also been reported previously in mangoes as well as in barks, kernels, peels and leaves [1,2,3,31,32].

The potential health benefits of various mango polyphenols are well documented in previous studies. For example, gallotannins and generated metabolites from intestinal microbial catabolism have been shown to possess anti-obesogenic effects in human and mouse models [4,33] and antibacterial effects in vitro [34,35]. Therefore, understanding the metabolic fate of mango polyphenols and their PK (pharmacokinetic) profiles after consumption will facilitate interpreting their roles in providing health benefits in humans.

### 2.2. Acute Study

The objective of the acute 24 h study was to characterize and determine PK parameters of mango polyphenols and the generated metabolites in human plasma after a single, one-time intake of a MP beverage. The acute study also aimed to evaluate the potential of VC to enhance absorption of MP polyphenols. The (poly)phenolic metabolites were identified using UHPLC-Q-TOF-MS in the pooled plasma (*n* = 13) at time points 0, 2, 8 and 24 h. A total of 18 (poly)phenolic metabolites were characterized in the pooled plasma samples at 2, 8 and 24 h after MP beverage intake (Table 1).

**Figure 1:**
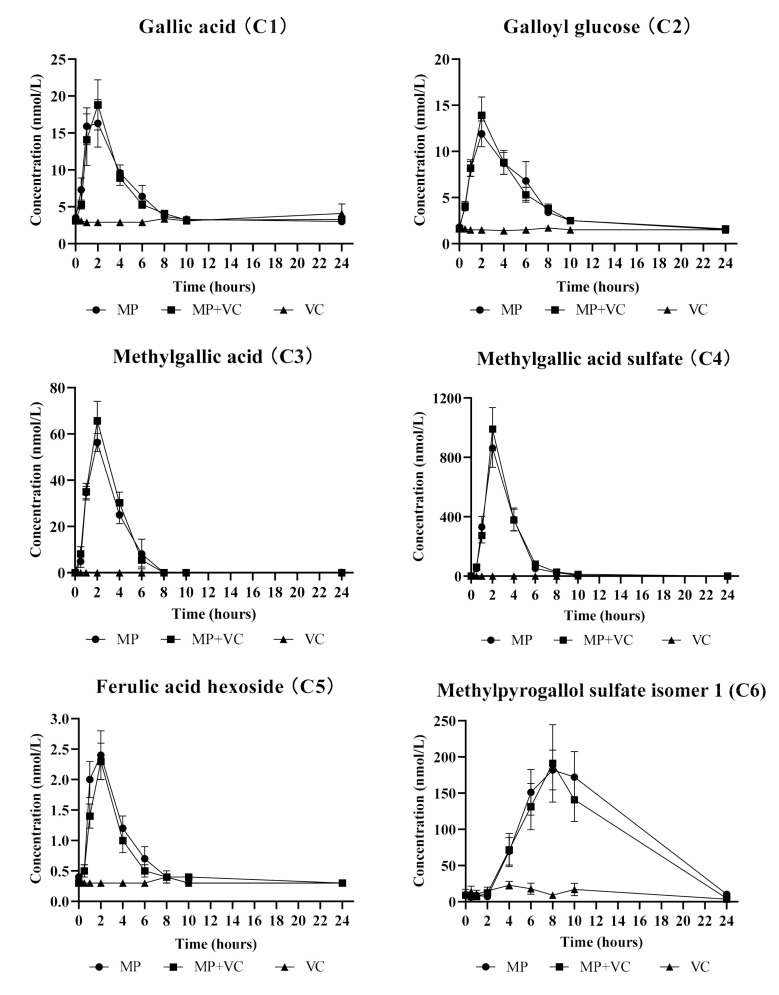

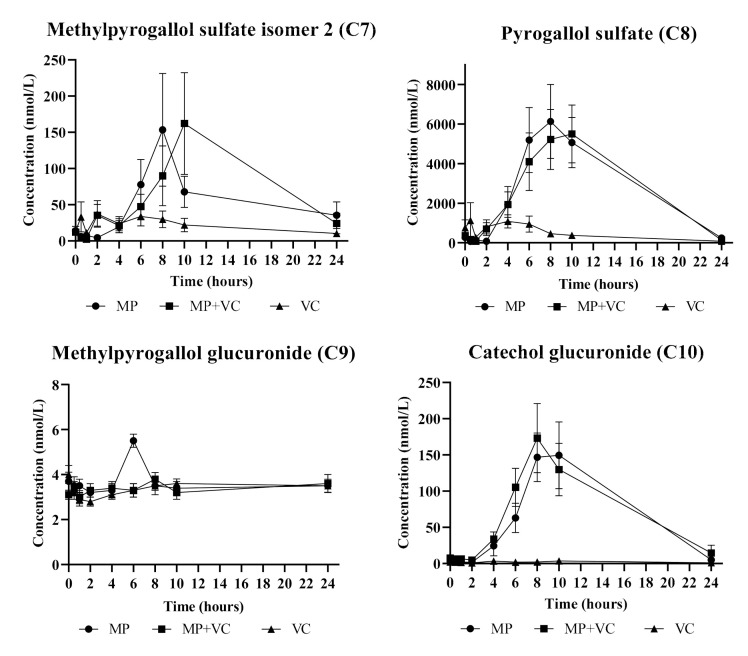
Plasma concentration vs. time profiles (0−24 h) of (poly)phenolic compounds and their metabolites: gallic acid (**C1**), galloyl glucose (**C2**), methylgallic acid (**C3**), methylgallic acid sulfate (**C4**), ferulic acid hexoside (**C5**), methylpyrogallol sulfate isomer 1 (**C6**), methylpyrogallol sulfate isomer 2 (**C7**), pyrogallol sulfate (**C8**), methylpyrogallol glucuronide (**C9**) and catechol glucuronide (**C10**), after test beverages intake. MP (mango pulp), MP + VC (mango pulp + vitamin C) and VC (vitamin C).

**Figure 2:**
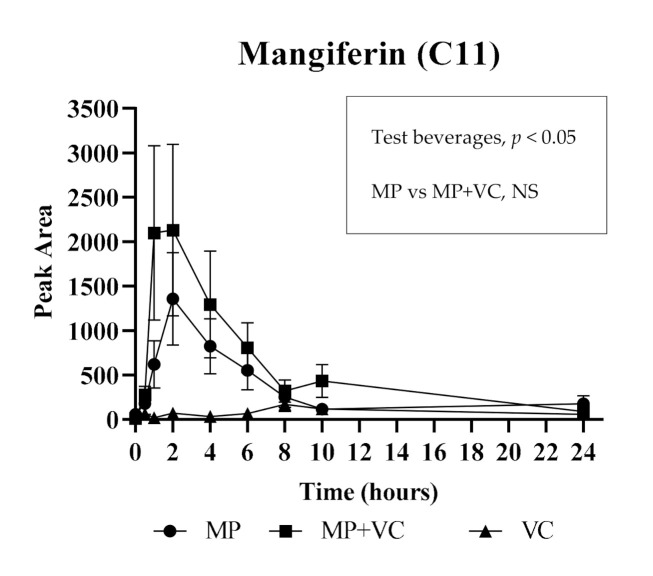
Area vs. time profile (0−24 h) of mangiferin (**C11**) after test beverages intake. The concentration of mangiferin was below the limit of quantitation (LOQ). MP (mango pulp), MP + VC (mango pulp + vitamin C) and VC (vitamin C). NS: not significant.

For the acute part of the study the quantitative analysis was conducted for 11 (poly)phenolic compounds and their metabolites in plasma. The mean plasma concentration vs. time profiles (0−24 h) are reported for 10 metabolites that were detected above the limit of quantitation (LOQ): gallic acid (**C1**), galloyl glucose (**C2**), methylgallic acid (**C3**), methylgallic acid sulfate (**C4**), ferulic acid hexoside (**C5**), methylpyrogallol sulfate isomer 1 (**C6**), methylpyrogallol sulfate isomer 2 (**C7**), pyrogallol sulfate (**C8**), methylpyrogallol glucuronide (**C9**) and catechol glucuronide (**C10**), after ingestion of MP, MP + VC and VC test beverages (Figure 1). The C_max_ and AUC_0–24h_ values of these 10 metabolites were significantly higher in plasma after MP and MP + VC beverages intake compared to VC beverage intake only except for methylpyrogallol glucuronide (**C9**) which failed the normality test for C_max_ during statistical analysis and had no significant differences in AUC_0–24h_ among treatments (Table 2). Mangiferin (**C11**) was detected but was below the LOQ. The pharmacokinetic curve of mangiferin is reported as the area vs. time profile (0−24 h) instead of mean plasma concentration vs. time profile (Figure 2). Mangiferin had significantly higher content in plasma (as observed from areas) after MP and MP + VC beverages intake compared to VC beverage intake alone (Table 2). The presence of gallic acid and mangiferin in plasma after MP beverage intake was confirmed by matching the MRM (multiple-reaction monitoring) ion transitions of the gallic acid and mangiferin standard with the compounds detected in the plasma. The identification of plasma metabolites including galloyl glucose, methylgallic acid, methylgallic acid sulfate, ferulic acid hexoside and methylpyrogallol glucuronide was confirmed by UHPLC-Q-TOF-MS and previous literature reports [2,20].

In general, (poly)phenolic compounds undergo structural modification before being absorbed into the blood. Compounds that escape absorption from the small intestine proceed to the colon where they are converted to various small molecules (phenolic acids, valerolactones, urolithins, etc.) by micro-organisms present in the lower bowel [36,37]. The metabolic products from the colon and the deconjugated (poly)phenols and aglycone structures from the upper digestive tract undergo phase II metabolism in the small intestine, liver and/or kidney resulting in methylated, glucuronidated and sulfoconjugated metabolites [38,39], while phase I metabolism (oxidation/reduction reactions) occurs to a lesser extent [40]. The resulting metabolites circulate in the blood and are transported to various body tissues and organs. Finally, the majority of metabolites are excreted in the urine by the kidneys [41].

Similar to mango pulp composition, most of the quantified compounds in plasma after acute supplementation of mango were gallic acid derivatives (Table 2, Figure 1 and Figure 2). Free gallic acid, galloyl glucose and gallic acid released from gallotannins were absorbed within 1–2 h and formed methyl, sulfate and glucuronide metabolites. Compounds such as gallic acid and its derivatives (**C1**–**C4**), ferulic acid hexoside (**C5**) and mangiferin (**C11**) reached their maximum concentration/area in plasma within 1–2 h after mango intake, and were cleared from the bloodstream within 6–8 h, suggesting absorption from the small intestine. On the other hand, several other metabolites (**C6**–**C10**) peaked at a much later time (8–10 h), suggesting microbial metabolism and absorption from the lower bowel. Pyrogallol is a major microbial metabolite of gallotannins [42] and gallic acid [43]. Several studies support a colonic origin of pyrogallol and its derivatives [21,33,44,45,46,47]. The conjugates of pyrogallol (**C6**–**C9**) achieved maximum concentrations in plasma between 6–10 h after mango intake. Catechol glucuronide (**C10**) showed a similar absorption pattern as pyrogallol derivatives. Inter-individual variability (high standard error) was observed in some metabolites especially those derived from gut microbial action possibly due to differences in the intestinal flora of individuals.

The enhancement of the absorption of mango polyphenols in the presence of VC was also explored in this acute study. There was an increase in the concentrations/areas of five metabolites, including gallic acid (**C1**), galloyl glucose (**C2**), methylgallic acid (**C3**), methylgallic acid sulfate (**C4**) and mangiferin (C11) after intake of MP + VC beverage compared to MP beverage alone (Figure 1 and Figure 2); however, the differences were not statistically significant (Table 2 and Figure 2). The polyphenols are prone to autoxidation when exposed to slightly basic environments such as the small intestine [48]. Vitamin C as an antioxidant may prevent the oxidation of mango polyphenols to some extent, such that simultaneous intake of mango with VC may reduce the gastrointestinal degradation of mango polyphenols. Previous studies have reported enhanced absorption of green tea polyphenols with VC. An increased absorption of epigallocatechin (EGC) and epigallocatechin gallate (EGCG) were observed in vitro [49], in vivo [50] and in humans [51] when green tea or its extracts were combined with VC-rich mixtures. In this study, even though higher concentrations/areas were observed for some metabolites in the MP + VC group compared to the MP group, the differences were not statistically significant. Further research with a higher dose of VC or combination with other bioactive compounds such as piperine [22] is needed to determine if dose or compound type could be the factors affecting absorption of (poly)phenolic compounds.

### 2.3. Short-Term 14 Day Mango Feeding Trial

In this part of the study, metabolite pool changes after daily intake of MP for 14 days were assessed. As noted in the acute study, several MP polyphenols are subjected to gut microbial metabolism. We hypothesized that with consistent daily intake/exposure to these polyphenols, an increase in generated metabolites would be observed due to an increase in microbial population or upregulation of the microbial mechanisms metabolizing the consistently available substrate.

A total of 166 (poly)phenolic metabolites were quantified in urine (Appendix A, Table A2). The compound which showed the highest urinary excretion when compared to fasting baseline urine was pyrogallol sulfate isomer (Δ = 1415.1 ± 955.2 nmol/L for 24 h fasting sample, Δ = 5298.0 ± 56.6 nmol/L for 15th day fasting sample). Similarly, Barnes et al. (2016) observed significantly increased excretion of pyrogallol sulfate in human urine after 10 days of MP intake (cv. Keitt, 400 g/day) [20]. Most of the compounds quantified in urine were detected in the plasma. However, we were not able to quantify them in the plasma due to their low concentrations (<LOQ).

**Figure 3:**
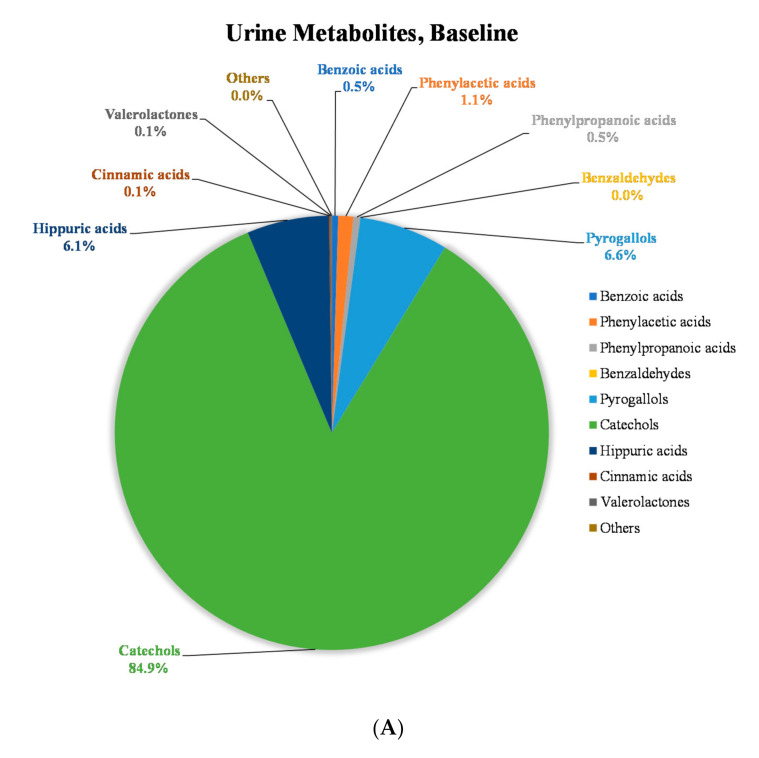

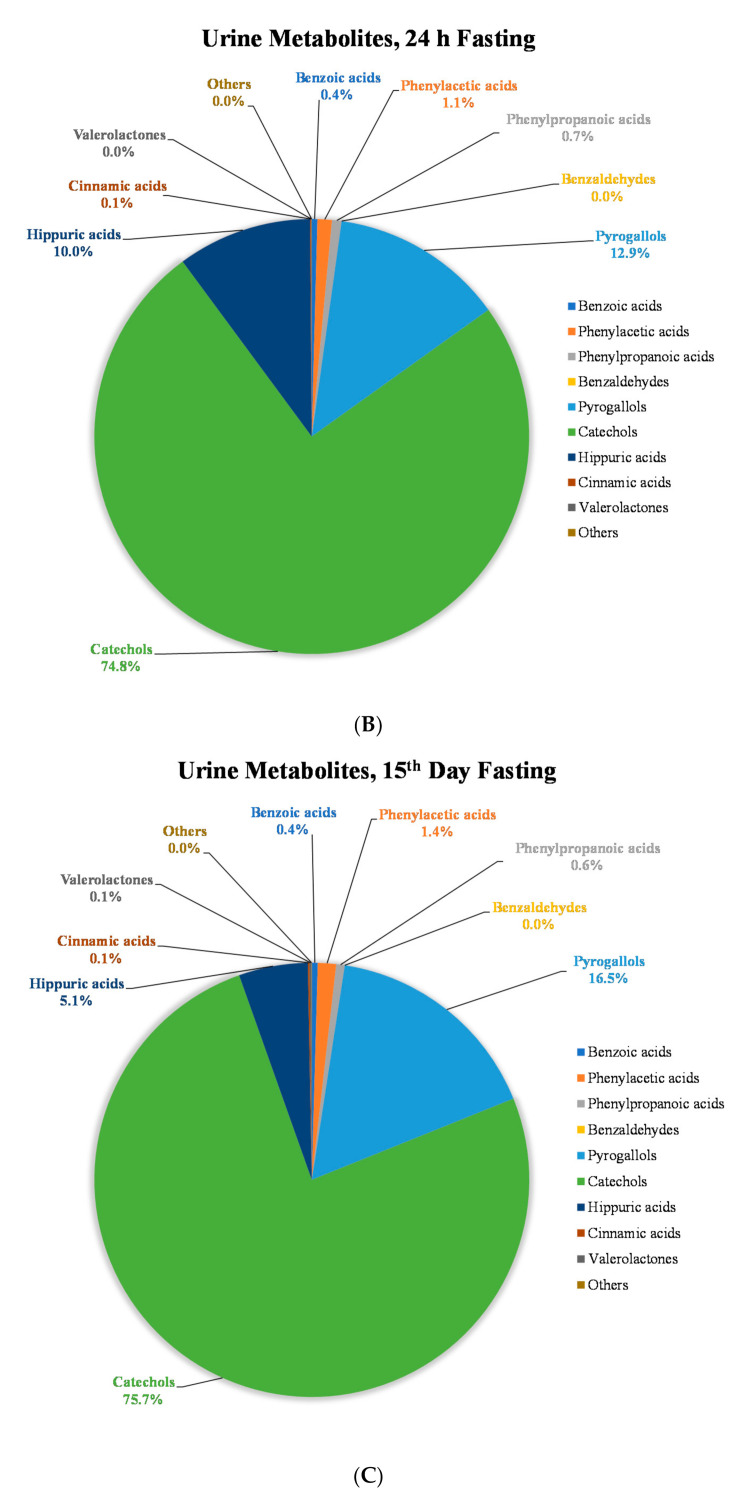
Distribution of concentration of (poly)phenolic metabolites classes in fasting urine at (**A**) baseline (0 h, before MP beverage intake), (**B**) 24 h fasting (24 h after single-time MP beverage intake) and (**C**) 15th day fasting (24 h after 14 days of MP beverage intake).

To get a better understanding of the changes in the polyphenolic metabolite pools after 14 days consumption of MP, the (poly)phenolic compounds quantified in urine samples were divided into 10 classes: benzoic acid derivatives, phenylacetic acid derivatives, phenylpropanoic acid derivatives, benzaldehyde derivatives, pyrogallol derivatives, catechol derivatives, hippuric acid derivatives, cinnamic acid derivatives, valerolactone derivatives and others (Appendix A, Table A2). The concentrations of polyphenol classes in urine were compared at baseline (0 h, before MP beverage intake), 24 h fasting (24 h after single-time MP beverage intake) and 15th day fasting (24 h after 14 days of MP beverage intake) (Figure 3, Appendix A, Table A2). The concentration increase of pyrogallol derivatives was highest in the 15th day fasting urine samples (3.6% increase) compared to 24 h fasting urine (*p* < 0.05). Minor increases (<1.0%) were also observed in other classes of compounds (Figure 3).

These results are in agreement with previous studies on mango conducted by the Talcott group where they observed significant (*p* < 0.05) increases of pyrogallol and benzoic acid derivatives in urine including methylpyrogallol sulfate, pyrogallol sulfate and methylgallic acid sulfate after consumption of MP [20,52,53]. The increased excretion of mango (poly)phenol (mainly gallotannins) metabolites could be due to the adaptive increase in microbial metabolism after 14 days of daily intake of mango polyphenols [4,33,54]. Generated metabolites support the growth of specific bacterial species, which in turn enhance the production of certain metabolites suggesting reciprocal interaction between them. Several gut microbial species have been associated with gallotannins catabolism. For example, colon microorganisms such as *Lactobacillus plantarum*, *Streptococcus galloylitcus*, *Aspergillus oryzae* and *Lactococcus lactis* can utilize gallotannins and produce gallic acid, pyrogallol and catechol [47,55]. Previous studies in humans demonstrated that repetitive mango supplementation can increase the abundance of pyrogallol-producing microbiota, e.g., *Aspergillus oryzae* and *Lactococcus lactis*, and decrease the abundance of *Bacteroides thetaiotaomicron* and *Clostridium leptum* [47,53]. Additionally, gallotannins and their metabolites may inhibit growth of other bacteria such as *Bacteroides fragilis*, *Escherichia coli*, *Enterobacter cloacae*, *Salmonella typhimurium*, *Salmonella aureus*, etc. [34,35,56], possibly increasing community space for blooming microbiota that can utilize gallotannins and increasing net metabolite concentrations. Enhanced microbial efficiency to metabolize polyphenols may also influence concentration of metabolites [57] although this may not be only true for mango polyphenols, but also for non mango-specific metabolites (i.e., hippuric acid).

### 2.4. Inter-Individual Variability

The inter-individual variability in (poly)phenolic compounds and their metabolites was calculated for baseline (0 h), 24 h fasting and 15th day fasting urine samples after MP beverage intake (Table 3). The percentage of the coefficient of variation (%CV) can show the extent of variability in relation to mean of the population. The highest inter-individual variability in terms of total polyphenols excreted in urine was observed at baseline (172%CV) which decreased tremendously after acute (64%CV) and 14 days (72%CV) of mango intake. The inter-individual variability was also determined in terms of classes of (poly)phenolic metabolites. Benzaldehyde derivatives (217%CV), pyrogallol derivatives (179%CV) and catechol derivatives (182%CV) had the highest %CV in urine at baseline (0 h) which decreased to 57%CV, 81%CV and 73%CV, respectively in 15th day fasting urine samples. On the other hand, the %CV of cinnamic acid derivatives, one of the major phenolic acid groups in mango pulp [58], increased from 68%CV to 136%CV after 14 days of daily intake of mango compared to baseline (Table 3).

Inter-individual variability has been documented previously in metabolite studies and may be attributed to a number of factors including but not limited to the differences in body weight/health status, the composition of individual gut microbiota [59,60], habitual dietary intake as these were free living individuals, host genetics including genetic polymorphism of cytochrome P450 enzymes resulting in an altered expression and function of individual enzymes, and work and living/lifestyle environments, among other influences [61,62].

In this study, we identified and quantified (poly)phenolic compounds in MP, as well as their metabolites after MP consumption in human plasma and urine with advanced instruments such as UHPLC-Q-TOF-MS and UHPLC-QQQ-MS. With our crossover design, we reduced the influence of confounding covariates because each subject served as their own control. However, the study has some limitations. The standards of most metabolites (glucuronides, sulfates etc.) were not commercially available at the time when the study was conducted, so those metabolites were quantified either with the respective parent compounds or with the standards that share similar structures or molecular weight with them. This may lead to differences between their actual concentrations and the reported values.

In summary, this study provides comprehensive characterization of (poly)phenolic metabolites generated after mango consumption in humans both in acute and repetitive intake settings. In addition, this is the first study to explore the effectiveness of the addition of VC for enhancing the absorption/metabolism of MP polyphenols. Understanding the metabolic fate and PK parameters of polyphenols from mangoes will aid in developing future research for assessing potential health benefits associated with mango consumption.

## 3. Materials and Methods

### 3.1. Ethics, Study Design and Study Subjects

**Figure 4:**
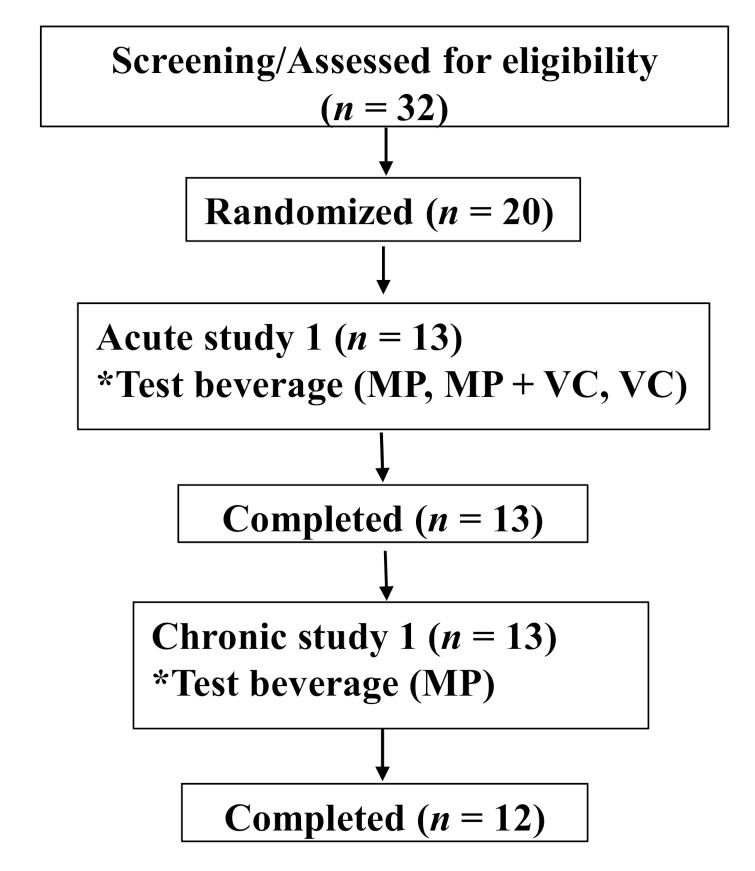
Consolidated Standards of Reporting Trials (CONSORT) flow diagram. * Test beverage: MP (Mango pulp (500 g)), MP+VC (Mango pulp (500 g) + Vitamin C (100 mg)) and VC (Vitamin C (100 mg)).

The research was conducted at the Clinical Nutrition Research Center (CNRC), Illinois Institute of Technology (IIT) (Chicago, IL, USA). The study was approved by the Institutional Review Board (IRB) and the trial was registered at clinicaltrials.gov (registration number: NCT03365739). All subjects signed the IRB approved informed consent form prior to the start of any study-related procedures. This was a two-part human clinical trial including an acute (24 h) and short-term (14 days) evaluation of MP intake. Thirteen healthy subjects were enrolled and completed both parts of the study protocol (Table 4, Figure 4). Subjects were nonsmokers and were not taking any supplements or medications (i.e., laxatives, proton pump inhibitors etc.) that would interfere with study outcomes. Subjects did not have documented atherosclerotic disease, digestive disorders, inflammatory disease, diabetes mellitus, or other systemic diseases. They did not have any allergy or intolerance to test beverages and study foods. Females of reproductive age were monitored, avoiding the menstruation phase of their menstrual cycle for study day visits.

#### 3.1.1. Acute 24 h Trial

**Figure 5:**
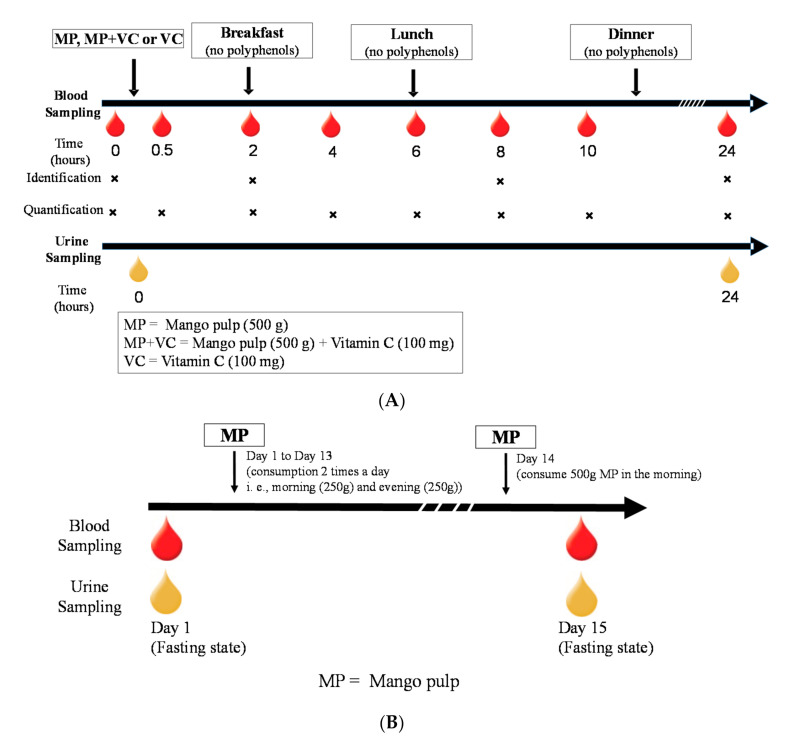
Blood and urine collection scheme on (**A**) an acute study day visit, (**B**) 14-day feeding trial.

The acute trial was a randomized, 3 arm, within-subject crossover study design (Figure 5A). The trial initiated with 3 days of food record collection to assess background (pre-study) dietary intake followed by counseling to adhere to a diet devoid of mango or its parts and relatively low in polyphenol-rich beverages/foods, which was maintained throughout the duration of the trial. Subjects were also provided a list of “Foods to Avoid” and alternate options to avoid high polyphenol foods and beverages, particularly for the 24 h before each study day, such as avoidance of coffee, tea, caffeinated products and alcohol. In general, subjects were encouraged to follow their “usual” diet with the exception of foods/beverages that may interfere with the outcomes of the study. Food intake diaries and, phone call and email reminders helped subjects maintain compliance throughout the study period. After an initial 7-day run-in period on the limited polyphenol diet, subjects were assigned to a randomization sequence providing 1 of 3 study test beverages (recipes provided in Appendix A, Table A3) on three separate days in a random order: MP (500 g), MP (500 g) + VC (100 mg) or VC (100 mg) beverages. On each study day subjects arrived fasted and well hydrated. After standard admission procedures were completed (e.g., review of compliance with dietary requirements for 3 days prior to study day, dinner meal intake, usual sleep patterns), a catheter was placed by a registered nurse in subjects’ non-dominant arm and a fasting/baseline blood sample was collected (0 h). Subjects were provided with a standard breakfast meal 2 h after the study beverages. The breakfast meal was comprised of a buttermilk biscuit with butter and jelly, and scrambled egg white with shredded white cheddar cheese. After the 6 h blood collection, subjects ate a low polyphenol lunch consisting of dry roasted peanuts and fresh peeled cucumber with ranch dressing (Appendix A, Table A3). Each visit lasted ~10.5 h and subjects were required to remain at the CNRC on the IIT Campus the entire time. After catheters were removed, subjects were given a controlled low polyphenolic dinner meal (provided at every study visit) to eat at home, with reminder instructions for overnight fasting and their scheduled time to report back to the CNRC the next morning for the 24 h blood and urine collection. Blood samples were collected at 0, 0.5, 2, 4, 6, 8, 10 and 24 h and urine samples were collected at 0 and 24 h (Figure 5A).

Blood samples were collected in vacutainers containing ethylenediaminetetraacetic acid (EDTA) and were centrifuged at 425 × *g* for 15 min at 4 °C to separate plasma. Spot urine samples were collected in urine collection cups, immediately placed on ice and then aliquoted into individual cryovials. All the samples were stored at −80 °C until analysis.

#### 3.1.2. Short-Term 14-Day Trial

A 14-day feeding trial was conducted with the MP only to study the effect of repetitive intake of MP on (poly)phenolic metabolite profiles. Upon completion of the acute trial, subjects were instructed to continue on the low polyphenolic diet for 2 weeks while consuming MP beverages (provided by CNRC) daily in the morning (250 g MP + 50 g water) and evening (250 g MP + 50 g water). On day 14, subjects consumed both beverages (500 g of MP in total) in the morning and reported to the CNRC on the 15th day after an overnight fast to provide a fasting blood and urine sample (Figure 5B). Blood and urine samples were collected as described above and stored at −80 °C until analysis.

### 3.2. Chemicals and Materials

HPLC-grade acetonitrile, methanol, acetone, formic acid, acetic acid and polypropylene syringe filters (Whatman^TM^ 0.2 µm) were purchased from Fischer Scientific Co. (Pittsburg, PA, USA). Solid Phase Extraction (SPE) cartridges (C18, 3 mL, 200 mg) were purchased from Agilent Technologies (Santa Clara, CA, USA). Standards of catechol (benzene-1,2-diol), hippuric acid, 2-hydroxyphenylacetic acid, 3-hydroxyphenylpropanoic acid, vanillic acid (4-hydroxy-3-methoxybenzoic acid), 3,4-dihydroxyphenylacetic acid, gallic acid (3,4,5-trihydroxybenzoic acid), ferulic acid (4’-hydroxy-3’-methoxycinnamic acid), 4-hydroxybenzoic acid, caffeic acid (3’,4’-dihydroxycinnamic acid), 2-methyhippuric acid, 4-methyhippuric acid, chlorogenic acid, 4-hydroxybenzaldehyde, 3,4-dihydroxybenzoic acid, 2,3-dihydroxybenzoic acid, 2,5-dihydroxybenzoic acid, *p*-coumaric acid (4’-hydroxycinnamic acid), *m*-coumaric acid (3’-hydroxycinnamic acid), *o*-coumaric acid (2’-hydroxycinnamic acid), catechin and mangiferin were purchased from Fischer Scientific Co. (Pittsburg, PA, USA). The metabolites (glucuronides, sulfates etc.) for which standards are not commercially available were quantified using the standards that share similar structures or molecular weight (the standards used to quantify each compound and their limit of quantification (LOQ) are listed in Appendix A, Table A4). Human blank plasma was purchased from BioIVT (San Francisco, CA, USA). Individually quick frozen (IQF) diced mangoes (variety: Kent) were purchased from Val-Mex Foods (San Antonio, TX, USA) and vitamin C powder (GMO Free Vitamins LLC) was purchased from Amazon.com.

### 3.3. Sample Preparation

#### 3.3.1. Extraction of (Poly)phenolic Compounds from MP

MP (5 g) was extracted with 20 mL of extraction solution (acetone: water: acetic acid = 70:29.7:0.3 *v*/*v*) followed by two subsequent extractions with 10 mL extraction solution. The samples were vortexed for 30 s, followed by 10 mins sonication in iced water. The samples were kept in the dark for 10 mins before centrifugation at 8228 g for 10 min at 4 °C. The supernatants were pooled together after three extractions and final volume was made up to 45 mL. Extract (1 mL) was dried under nitrogen gas and reconstituted in 2 mL of acidified water (0.1% formic acid) for SPE procedure. The elution of the compounds was done with 1 mL of acidified methanol (0.1% formic acid). The eluent was collected and dried under nitrogen gas at room temperature. The dried samples were reconstituted in 200 µL of starting mobile phase (SMP) (0.1% formic acid, 5% acetonitrile in water) and centrifuged at 18,514 × *g* for 10 min at 4 °C. Supernatant was collected in amber HPLC vials before analysis.

#### 3.3.2. Extraction of (Poly)phenolic Compounds from Plasma

SPE procedure was conducted to extract and concentrate the (poly)phenolic compounds and their metabolites from the plasma samples. Briefly, plasma samples were thawed on ice and 500 µL plasma was diluted with 1.5 mL of 0.1% formic acid in water with addition of internal standards (2-methylhippuric acid (2 ng/mL), phloridizin (1 ng/mL)) before being loaded on preconditioned SPE cartridges. The cartridges were washed with 0.1% formic acid in water (1 mL) after loading with plasma samples. The elution of the compounds was achieved by 0.1% formic acid in methanol (1 mL) and the eluent was dried under nitrogen gas at room temperature. The dried samples were reconstituted in 100 µL of SMP and centrifuged at 18,514× *g* for 10 mins at 4 °C. Supernatant was collected in amber HPLC vials before analysis.

#### 3.3.3. Extraction of (Poly)phenolic Compounds from Urine

Urine (500 µL) was diluted and centrifuged at 18,514 × *g* for 10 mins at 4 °C. The supernatant was collected and filtered through 0.2 µm polypropylene syringe filter before HPLC analysis.

### 3.4. Identification and Quantification of (Poly)phenolic Compounds

The (poly)phenolic compounds were identified using an Agilent 1290 Infinity ultrahigh-performance liquid chromatography (UHPLC) system coupled with Agilent 6550 electrospray ionization (ESI) quadrupole time of flight (Q-TOF) mass spectrometer (MS) (Agilent Technologies, Santa Clara, CA, USA). The system was equipped with a binary pump with an integrated vacuum degasser, autosampler with a thermostat and column compartment with a thermostat. Spectra were recorded in negative mode with the following parameters: gas temperature 250 °C, gas flow 10 L/min, nebulizer pressure 35 psi, sheath gas temperature 300 °C, sheath gas flow 11 L/min and capillary 3500 V. MS scan method and Auto MS preferred list of the compounds was created based on personal compound database library (PCDL) and literature reports. Spectra were acquired in MS scan with the *m*/*z* range of 100–1200 and an acquisition rate of 2 spectra per s, and in MS/MS mode with the *m/z* range of 50–1200 and an acquisition rate of 4 spectra per s. The compound identification was based on the MS/MS fragmentation pattern, exact mass (PCDL), retention time match with available standards and previous literature reports. Compounds with mass error less than 5 ppm and/or retention time match with the standards were considered for identification. The data were analyzed using the Mass Hunter Qualitative Analysis software (version B.06.00, Agilent Technologies, Santa Clara, CA, USA). Pooled plasma (*n* = 13) samples were analyzed at 0, 2, 8 and 24 h for identification of (poly)phenolic metabolites.

A UHPLC system coupled with a 6460 Series Triple Quadrupole (QQQ) (Agilent Technologies, Santa Clara, CA, USA) was used for quantitative analysis. The ESI conditions were the same as those used in the UHPLC-Q-TOF analysis. The quantification of the compounds was conducted by multiple-reaction monitoring (MRM) transitions. Spectra were recorded in both positive- and negative-ion mode with capillary voltage of 4500 V and drying gas flow rate of 9 L/min at 200 °C. The sheath gas temperature and flow rate were 300 °C and 11 L/min, respectively. Standards were optimized for collision energies, fragmentor voltages and MRM transitions using Mass Hunter Optimizer. The MRM transition for metabolites were created based on the results from UHPLC-Q-TOF-MS. Standards for plasma analysis were prepared in blank plasma (charcoal-stripped human plasma obtained from Bioreclamation IVT) for matrix match and for urine analysis were prepared in SMP.

(Poly)phenolic compounds and their metabolites in plasma and urine were separated on a Pursuit 3 PFP column (150 × 2.0 mm, 3.0 μm, Agilent Technologies) equipped with a Pursuit MetaGuard column (10 × 2.0 mm, 3.0 μm, Agilent Technologies) at a constant temperature of 35 °C using our previously established method [29]. The flow rate was maintained at 0.4 mL/min and the injection volume was 5 μL. The mobile phase consisted of acidified water (0.1% formic acid) (A) and acidified acetonitrile (0.1% formic acid) (B). The gradient for the separation of compounds was as follows: 5 to 5% B from 0 to 1 min; 10% B at 3 min; 15% B at 7 min; 15% B at 9 min; 20% B at 10 min; 20% B at 11 min; 25% B at 12 min; 30% B at 13 min; 30% B at 14 min; 95% B at 15 min; and back to 5% B at 16 min. The column was re-equilibrated to the initial mobile phase conditions for 4 min before the next injection. The data were analyzed using the Mass Hunter Quantitative Analysis software (version B.07.00, Agilent Technologies, Santa Clara, CA, USA).

### 3.5. Pharmacokinetic and Statistical Analysis

The absorption and elimination of 11 major mango metabolites over 24 h was determined after MP, MP + VC, and VC intake. The plasma concentration/area at each time point was determined by the mean of 13 subjects. The time vs. concentration curves and the time vs. area curves were created using GraphPad Prism (version 8.2.1). C_max_ (nmol/L) is defined as the maximum concentration of (poly)phenolic metabolites in plasma between 0−24 h after test beverage intake. T_max_ is the time (h) when C_max_ was achieved. Wilcoxon signed-rank test was used for T_max_ analysis due to non-normal distribution. Area under the plasma concentration time curve (AUC_0−24h_, nmol·h/L) was calculated by the linear trapezoidal method using Microsoft Excel 2013, version 15 [63].

Subject characteristics were analyzed from data collected at the screening visit and tabulated using descriptive statistics. Results are presented as numbers and percentages, as appropriate. For continuous variables, normality was assessed using Shapiro–Wilk tests, skewness and kurtosis. Data not conforming to normal distribution patterns were log transformed prior to analysis and noted accordingly. Mixed-model repeated measure analysis of covariance (ANCOVA) was performed on each quantitative outcome variable to test main effects of test beverages and time (hour) using PROC MIXED via Window PC-SAS (version 9.4; SAS Institute Inc, Cary, NC). Multiple comparisons within and among test beverages (MP, MP + VC and VC) over 24 h were performed by mixed model statistical significance (*p* < 0.05). A two-tailed distribution, paired t-test (Microsoft Excel 2013, version 15) was conducted for a short-term 14-day feeding trial to compare the (poly)phenolic metabolite concentrations in fasting urine collected after acute (24 h) and 14-day MP intake (15th day). The pharmacokinetic (PK) curves of the (poly)phenolic metabolites with plasma concentrations below LOQ are presented as area vs. time instead of area vs. concentration curves.

The results of the statistical analysis are presented as mean ± standard error unless indicated otherwise. Statistical significance was based on 2-sided test beverage comparison at the 5% significance level under a null hypothesis of no difference between test beverages.

## Figures and Tables

**Table 1:** (Poly)phenolic metabolites tentatively identified in plasma at different time points after mango pulp beverage intake (acute trial).

	Compound Number	Retention Time (min)	Observed Ion (*m*/*z*)	Calculated (*m*/*z*)	Mass Error	Molecular Formula	Tentative Compound Identification
2 h	1	1.2	367.10464	367.10287	4.8	C_17_H_20_O_9_	Feruloylquinic acid
	2	4.5	183.02976	183.02937	2.1	C_8_H_8_O_5_	Methylgallic acid
	3	4.7	167.03452	167.03447	0.3	C_8_H_8_O_4_	Vanillic acid
	4	6.5	285.06163	285.06107	2.0	C_12_H_14_O_8_	Catechol glucuronide
	5	9.2	299.07736	299.07667	2.3	C_13_H_16_O_8_	Hydroxybenzoic acid glucoside
	6	14.2	471.18600	471.18667	−1.4	C_22_H_32_O_11_	Abscisic acid glucose ester (formate adduct)
	7	14.4	192.06678	192.06607	3.7	C_10_H_11_NO_3_	Methylhippuric acid
8 h	1	4.5	315.07213	315.07157	1.8	C_13_H_16_O_9_	Methylpyrogallol glucuronide
	2	5.3	371.09754	371.09787	−0.9	C_16_H_20_O_10_	Methoxyphenylpropanoic acid glucuronide
	3	6.0	204.98140	204.98067	3.6	C_6_H_6_O_6_S	Pyrogallol sulfate
	4	8.9	218.99631	218.99637	−0.3	C_7_H_8_O_6_S	Methylpyrogallol sulfate
	5	9.3	299.07739	299.07667	2.4	C_13_H_16_O_8_	Hydroxybenzoic acid glucoside
	6	22.1	331.0668	331.06657	3.7	C_13_H_16_O_10_	Galloyl glucose
24 h	1	5.3	194.04533	194.04537	−0.2	C_9_H_9_NO_4_	Hydroxyhippuric acid
	2	6.4	341.08881	341.08727	4.5	C_15_H_18_O_9_	Hydroxyphenylproanoic acid glucuronide
	3	7.9	218.99631	218.99637	−0.3	C_7_H_8_O_6_S	Methylpyrogallol sulfate
	4	15.9	194.04602	194.04537	3.3	C_9_H_9_NO_4_	Hydroxyhippuric acid isomer
	5	20.7	597.10752	597.10917	−2.8	C_25_H_26_O_17_	Iso/mangiferin glucuronide

These metabolites were not observed at time point *t* = 0 h (baseline i.e., before mango intake).

**Table 2:** Pharmacokinetic parameters (0−24 h) and *p* values of (poly)phenolic compounds and their metabolites in human plasma after ingestion of MP, MP + VC and VC test beverages (mean ± standard error).

Compound Names (Number)	Pharmacokinetic Parameters	MP (A)	MP + VC (B)	VC (C)	*p* Value			
					Tx	A vs. B	A vs. C	B vs. C
Gallic acid (**C1)**	C_max_ (nmol/L)	20.4 ± 3.1	21.1 ± 3.9	5.3 ± 1.3	<0.0001	NS	<0.0001	<0.0001
	T_max_ (h)	2.2 ± 0.4	2.0 ± 0.3	N/A		NS	N/A	N/A
	AUC_0–24h_ (nmol·h/L)	64.5 ± 7.8	62.8 ± 6.9	24.9 ± 1.9	<0.0001	NS	<0.0001	<0.0001
Galloyl glucose (**C2**)	C_max_ (nmol/L)	13.4 ± 1.9	14.0 ± 2.0	2.1 ± 0.3	<0.0001	NS	<0.0001	<0.0001
	T_max_ (h)	2.4 ± 0.3	2.2 ± 0.2	N/A		NS	N/A	N/A
	AUC_0–24h_ (nmol·h/L)	46.1 ± 5.0	48.0 ± 5.6	12.3 ± 0.7	<0.0001	NS	<0.0001	<0.0001
Methylgallic acid (**C3**)	C_max_ (nmol/L)	54.5 ± 4.9	65.7 ± 8.4	4.8 ± 0.7	<0.0001	NS	<0.0001	<0.0001
	T_max_ (h)	2.2 ± 0.3	2.0 ± 0.0	N/A		NS	N/A	N/A
	AUC_0–24h_ (nmol·h/L)	161.1 ± 13.6	178.7 ± 17.5	25.9 ± 1.9	<0.0001	NS	<0.0001	<0.0001
Methylgallic acid sulfate (**C4**)	C_max_ (nmol/L)	824.2 ± 130.8	990.8 ± 145.0	6.3 ± 1.6	<0.0001	NS	<0.0001	<0.0001
	T_max_ (h)	2.2 ± 0.2	2.0 ± 0.0	N/A		NS	N/A	N/A
	AUC_0–24h_ (nmol·h/L)	1655.8 ± 274.0	1837.1 ± 258.3	24.7 ± 3.0	<0.0001	NS	<0.0001	<0.0001
Ferulic acid hexoside (**C5**)	C_max_ (nmol/L)	2.3 ± 0.4	2.4 ± 0.3	0.5 ± 0.1	<0.0001	NS	<0.0001	<0.0001
	T_max_ (h)	1.8 ± 0.2	1.8 ± 0.1	N/A		NS	N/A	N/A
	AUC_0–24h_ (nmol·h/L)	7.7 ± 1.2	6.8 ± 0.7	2.5 ± 0.1	<0.0001	NS	<0.0001	<0.0001
Methylpyrogallol sulfate isomer (**C6**)	C_max_ (nmol/L)	230.2 ± 32.3	224.9 ± 50.3	35.8 ± 9.5	<0.0001	NS	<0.0001	<0.0001
	T_max_ (h)	8.6 ± 0.5	7.7 ± 0.6	N/A		NS	N/A	N/A
	AUC_0–24h_ (nmol·h/L)	616.9 ± 83.7	578.1 ± 103.7	129.5 ± 36.0	<0.0001	NS	<0.0001	<0.0001
Methylpyrogallol sulfate isomer (**C7**)	C_max_ (nmol/L)	243.5 ± 71.8	232.8 ± 69.8	76.6 ± 24.9	<0.05	NS	<0.05	<0.05
	T_max_ (h)	9.8 ± 1.8	6.3 ± 1.0	N/A		NS	N/A	N/A
	AUC_0–24h_ (nmol·h/L)	375.4 ± 94.3	394.6 ± 89.1	216.6 ± 54.5	<0.05	NS	<0.05	<0.05
Pyrogallol sulfate (**C8**)	C_max_ (nmol/L)	10023.8 ± 1781.1	8823.4 ± 1767.4	2564.2 ± 893.5	<0.05	NS	<0.05	<0.05
	T_max_ (h)	7.3 ± 0.6	7.3 ± 0.7	N/A		NS	N/A	N/A
	AUC_0–24h_ (nmol·h/L)	18852.1 ± 3491.6	17956.9 ± 3093.6	5504.9 ± 1763.4	<0.05	NS	<0.05	<0.05
Methylpyrogallol glucuronide (**C9**)	C_max_ (nmol/L)	6.7 ± 2.3	4.5 ± 0.3	4.8 ± 0.3	F	F	F	F
	T_max_ (h)	7.2 ± 2.4	9.7 ± 2.4	8.0 ± 2.3		NS	N/A	N/A
	AUC_0–24h_ (nmol·h/L)	29.1 ± 3.0	26.7 ± 1.8	26.1 ± 1.7	NS	NS	NS	NS
Catechol glucuronide (**C10**)	C_max_ (nmol/L)	471.7 ± 270.2	185.8 ± 45.8	10.6 ± 3.2	<0.0001	NS	<0.0001	<0.0001
	T_max_ (h)	8.0 ± 0.6	8.3 ± 0.4	N/A		NS	N/A	N/A
	AUC_0–24h_ (nmol·h/L)	1830.9 ± 1391.0	483.3 ± 108.0	44.8 ± 5.1	<0.0001	NS	<0.0001	<0.0001

Test beverages—A: MP (mango pulp), B: MP + VC (mango pulp + vitamin C), C: VC (vitamin C). Tx: test beverage effect, NS: not significant, N/A: not applicable, F: failed normality test.

**Table 3:** Coefficient of variation (%CV) of (poly)phenolic metabolites in fasting urine samples after acute and 14 days of mango beverage consumption.

	0 h	24 h	Day 15
Benzoic acid derivatives	47	49	65
Phenylacetic acid derivatives	59	51	71
Phenylpropanoic acid derivatives	48	57	76
Benzaldehyde derivatives	217	98	57
Pyrogallol derivatives	179	74	81
Catechol derivatives	182	64	73
Hippuric acid derivatives	61	50	36
Cinnamic acid derivatives	68	38	136
Valerolactone derivatives	112	117	112
Others	85	47	51
Total polyphenols	172	64	72

Coefficient of variation (%CV) (standard deviation/mean concentration × 100%).

**Table 4:** Subject demographic characteristics.

Variable	Acute Trial (*n* = 13)	14-Day Feeding Trial (*n* = 12)
Age (year)	30 ± 2	31 ± 2
Height (cm)	169.5 ± 2.1	169.4 ± 2.2
Weight (kg)	64.9 ± 1.9	65.0 ± 2.0
BMI (kgm^−2^)	22.7 ± 0.4	22.7 ± 0.5
Heart rate (beats per minute)	68 ± 2	69 ± 2
Systolic blood pressure (mmHg)	115 ± 2	115 ± 2
Diastolic blood pressure (mmHg)	73 ± 2	74 ± 2
Mid-point waist circumference (cm)	78.8 ± 1.1	78.8 ± 1.2
Male:Female	7:6	6:6

Values are presented as mean ± standard error.

## Appendix A

**Table A1:** (Poly)phenolic compounds tentatively identified in IQF (individually quick frozen) mango pulp using UHPLC-Q-TOF mass spectrometry.

Compound Number	Retention Time (min)	Precursor Ion (*m/z*)	Calculated (*m*/*z*)	Mass Error	Molecular Formula	Product Ion (*m*/*z*)	Tentative Compound Identification
1	1.3	191.05544	191.05556	−0.6	C_7_H_12_O_6_	191.05575, 129.04033, 145.10961	Quinic acid *
2	1.5	191.01904	191.01917	−0.7	C_6_H_8_O_7_	111.00075, 129.70383	Citric acid
3	2.1	493.11906	493.11934	−0.6	C_19_H_26_O_15_	169.01264, 313.05263, 405.25778	Galloyl diglucoside
4, 5	2.4, 2.8	331.06624	331.06652	−0.8	C_13_H_16_O_10_	125.02369, 169.01343	Galloyl glucose
6	2.9	153.01858	153.01878	−1.3	C_7_H_6_O_4_	125.02388, 136.94855, 108.99133	Protocatechuic acid (3,4-Dihydroxybenzoic acid) *
7	3.0	167.03402	167.03443	−2.5	C_8_H_8_O_4_	152.00786, 132.86693, 110.59943	Vanillic acid *
8	3.1	343.06588	343.06652	−1.9	C_14_H_16_O_10_	168.91982, 191.05969	Galloylquinic acid
9	3.3	299.07664	299.07669	−0.2	C_13_H_16_O_8_	137.02997, 179.03352	Hydroxybenzoic acid glucoside
10	3.9	179.03412	179.03443	−1.7	C_9_H_8_O_4_	135.04455, 118.93691	Caffeic acid (3,4-Dihydroxycinnamic acid) *
11	3.9	341.08707	341.08725	−0.5	C_15_H_18_O_9_	135.04457, 179.03434	Caffeoyl glucose
12	4.0	325.09221	325.09234	−0.4	C_15_H_18_O_8_	119.04938, 163.03948	Coumaroyl hexoside
13	4.0	163.03947	163.03951	−0.2	C_9_H_8_O_3_	163.03878, 119.04942	Coumaric acid
14, 15, 16, 17, 18, 19	4.1, 4.7, 4.9, 5.6, 7.0	635.08856	635.08843	−0.2	C_27_H_24_O_18_	169.01003, 465.06588	Trigalloyl glucose
	4.5, 6.7	443.19130	443.19172	−0.9	C_21_H_32_O_10_	101.02350, 113.02310	Dihydrophaseic acid glucoside
20	4.7	137.02392	137.02386	−0.4	C_7_H_6_O_3_	136.95173, 65.0389, 93.03008	Hydroxybenzoic acid
21	4.7	193.04965	193.05008	−2.2	C_10_H_10_O_4_	134.03702, 178.02565, 149.05928	Ferulic acid *
22	4.7, 5.9	355.10269	355.10290	−0.6	C_16_H_20_O_9_	134.03661, 193.04963, 178.02627	Ferulic acid hexoside
23	5.0	289.07077	289.07121	−1.5	C_15_H_14_O_6_	109.02822, 245.08098, 203.06967	Catechin *
24	5.4	223.05998	223.06064	−3.0	C_11_H_12_O_5_	123.90067, 193.01364, 135.04564	Sinapic acid *
25, 26, 27, 28, 29, 30	5.7, 6.1, 6.6, 7.0, 7.5, 7.7	403.16012	403.16042	−0.7	C_21_H_24_O_8_	197.11786, 241.10404	Hydroxy-dimethyl decadiene-dioic acid glucopiranosylester
31	6.1	421.07560	421.07708	−3.5	C_19_H_18_O_11_	331.04450, 301.03487	Mangiferin
32	6.5	447.09407	447.09273	3.0	C_21_H_20_O_11_	169.01225, 284.95930	Kaempferol 3-*O*-glucoside *
33	6.7	353.08664	353.08725	−1.7	C_16_H_18_O_9_	173.13091	Caffeoyl quinic acid (chlorogenic acid) *
34	6.8	477.10296	477.10330	−0.7	C_22_H_22_O_12_	315.05587, 163.03885, 119.04845	Isorhamnetin hexoside
35, 36, 37, 38, 39	6.8, 7.1, 8.0, 8.5, 8.9	787.09950	787.09939	−0.1	C_34_H_28_O_22_	169.01361, 393.34982	Tetragalloyl glucose
40	9.2	300.99786	300.99844	−1.9	C_14_H_6_O_8_	145.02748, 229.01413, 283.99356	Ellagic acid *
41	9.5	449.10773	449.10838	−1.4	C_21_H_22_O_11_	151.00166, 227.12791, 287.05559	Eriodictyol hexoside
42	9.9	463.08719	463.08765	−1.0	C_21_H_20_O_12_	300.02418, 301.03161, 271.02316	Quercetin−3-*O*-glucoside *
43	10.1	471.18617	471.18663	−1.0	C_22_H_32_O_11_	153.09122, 263.13877, 219.13877	Abscisic acid glucose ester (formate adduct)
44, 45	10.2, 16.0	263.12825	263.12833	−0.3	C_15_H_20_O_4_	153.09088, 219.13969, 245.84075	Abscisic acid
46	11.0	939.11025	939.11035	−0.1	C_41_H_32_O_26_	169.01349, 617.07628, 769.08941	Pentagalloyl glucose

All these compounds were tentatively identified based on the Agilent’s personal compound database library (mass error < 5 ppm). * Identity of compounds confirmed by standards.

**Table A2:** Concentration of (poly)phenolic compounds and their metabolites in fasting urine samples: baseline (0 h, before MP beverage intake), 24 h fasting (24 h after single-time MP beverage intake) and 15th day fasting (24 h after 14-day MP beverage intake).

Compounds	Retention Time	Mean ± std Error (µmol/L)			*p* Value	Transition
	(min)	0 h (Fasting)	24 h (Fasting)	15 Days (Fasting)	24 h vs. 15 Days	(*m*/*z*)
**Benzoic acid Derivatives**						
* 3,4-Dihydroxybenzoic acid ^q^	4.2	2.4 ± 0.7	1.1 ± 0.3	0.8 ± 0.2	NS	153.0 → 109.0
* 2,5-Dihydroxybenzoic acid ^r^	5.8	6.7 ± 1.6	4.9 ± 1.0	7.9 ± 2.7	NS	153.0 → 109.0
* 2,3-Dihydroxybenzoic acid ^s^	6.5	0.3 ± 0.1	0.2 ± 0.1	0.3 ± 0.1	NS	153.0 → 109.0
Dihydroxybenzoic acid sulfate isomer 1 ^t^	3.5	1.3 ± 0.5	0.7 ± 0.2	2.3 ± 1.0	NS	233.0 → 153.0
Dihydroxybenzoic acid sulfate isomer 2 ^t^	4.2	18.3 ± 4.7	8.1 ± 1.8	12.8 ± 3.3	NS	233.0 → 153.0
Dihydroxybenzoic acid sulfate isomer 3 ^t^	5.0	5.8 ± 1.8	2.7 ± 0.6	Not detected	N/A	233.0 → 153.0
Dihydroxybenzoic acid sulfate isomer 4 ^t^	6.6	1.1 ± 0.7	1.0 ± 0.3	1.3 ± 0.2	NS	233.0 → 153.0
* 4-Hydroxybenzoic acid ^a^	5.4	6.1 ± 1.0	5.9 ± 0.9	5.4 ± 0.8	NS	137.0 → 93.0
* Hydroxybenzoic acid 1 ^a^	5.0	3.8 ± 0.8	2.7 ± 0.5	2.4 ± 0.7	NS	137.0 → 93.0
* Hydroxybenzoic acid 2 ^a^	4.4	3.4 ± 1.7	1.3 ± 0.1	2.6 ± 1.8	NS	137.0 → 93.0
* Hydroxybenzoic acid 3 ^a^	10.9	1.9 ± 0.3	2.9 ± 0.6	3.2 ± 1.5	NS	137.0 → 93.0
Hydroxybenzoic acid hexoside ^a^	4.4	0.6 ± 0.0	0.5 ± 0.0	0.2 ± 0.0	NS	299.1 → 137.0
Hydroxybenzoic acid sulfate isomer 1 ^a^	4.5	6.9 ± 4.3	2.5 ± 0.5	6.3 ± 4.2	NS	217.0 → 137.0
Hydroxybenzoic acid sulfate isomer 2 ^a^	11.7	5.0 ± 1.4	7.0 ± 3.6	16.7 ± 7.0	NS	217.0 → 137.0
Hydroxybenzoic acid glucuronide isomer 1 ^a^	3.7	0.6 ± 0.0	0.4 ± 0.0	0.3 ± 0.0	<0.05	313.1 → 137.0
Hydroxybenzoic acid glucuronide isomer 2 ^a^	4.7	1.0 ± 0.2	0.8 ± 0.1	0.8 ± 0.4	NS	313.1 → 137.0
Hydroxybenzoic acid glucuronide isomer 3 ^a^	6.6	0.9 ± 0.1	0.7 ± 0.1	0.6 ± 0.2	NS	313.1 → 137.0
Hydroxybenzoic acid glucuronide isomer 4 ^a^	8.0	0.5 ± 0.0	0.4 ± 0.0	0.2 ± 0.0	<0.05	313.1 → 137.0
* Vanillic acid (4-Hydroxy-3-methoxybenzoic acid) ^b^	6.4	4.9 ± 0.9	4.4 ± 0.7	4.1 ± 1.0	NS	167.0 → 152.0
* Hydroxymethoxybenzoic acid isomer 1 ^b^	5.6	4.6 ± 0.7	3.4 ± 0.3	5.5 ± 1.2	NS	167.0 → 152.0
Methoxybenzoic acid glucuronide isomer 1 ^d^	1.3	14.0 ± 8.0	7.4 ± 3.0	2.7 ± 0.6	NS	343.1 → 167.0
Methoxybenzoic acid glucuronide isomer 2 ^d^	3.6	2.8 ± 0.7	1.4 ± 0.2	2.2 ± 0.6	NS	343.1 → 167.0
Methoxybenzoic acid glucuronide isomer 3 ^d^	4.3	5.8 ± 2.7	2.9 ± 0.6	7.3 ± 2.5	NS	343.1 → 167.0
Methoxybenzoic acid sulfate isomer 1 ^d^	3.7	132.2 ± 26.0	59.4 ± 12.3	77.2 ± 23.2	NS	247.0 → 167.0
Methoxybenzoic acid sulfate isomer 2 ^d^	5.7	43.7 ± 14.3	33.6 ± 76	45.7 ± 10.9	NS	247.0 → 167.0
Methoxybenzoic acid sulfate isomer 3 ^d^	7.2	2.0 ± 0.5	0.8 ± 0.1	1.1 ± 0.2	NS	247.0 → 167.0
Methoxybenzoic acid sulfate isomer 4 ^d^	9.0	5.7 ± 1.6	4.3 ± 0.7	4.3 ± 2.4	NS	247.0 → 167.0
* Gallic acid (3,4,5-Trihydroxybenzoic acid) ^c^	2.4	0.2 ± 0.0	0.2 ± 0.0	0.1 ± 0.0	<0.05	169.0 → 125.0
Gallic acid derivative isomer 1 ^c^	9.0	0.3 ± 0.1	0.4 ± 0.2	1.0 ± 0.3	NS	311.0 → 125.0
Gallic acid derivative isomer 2 ^c^	12.0	0.1 ± 0.0	0.1 ± 0.0	0.2 ± 0.1	NS	311.0 → 125.0
Galloylshikimic acid isomer 1 ^c^	1.7	0.6 ± 0.1	0.4 ± 0.1	0.3 ± 0.1	NS	325.1 → 125.0
Galloylshikimic acid isomer 2 ^c^	2.4	0.5 ± 0.1	0.2 ± 0.0	0.4 ± 0.1	NS	325.1 → 125.0
Galloylshikimic acid isomer 3 ^c^	4.7	0.4 ± 0.2	0.5 ± 0.1	0.8 ± 0.2	NS	325.1 → 125.0
Galloylshikimic acid isomer 4 ^c^	9.7	0.1 ± 0.0	0.1 ± 0.1	0.4 ± 0.1	NS	325.1 → 125.0
Galloyl glucose isomer 1 ^c^	1.3	0.2 ± 0.0	0.1 ± 0.0	0.1 ± 0.0	NS	331.1 → 125.0
Galloyl glucose isomer 2 ^c^	3.5	0.1 ± 0.0	0.1 ± 0.0	0.2 ± 0.1	NS	331.1 → 169.0
Methyl gallate isomer 1 ^c^	4.6	0.6 ± 0.1	0.5 ± 0.1	2.2 ± 1.1	NS	183.2 → 124.0
Methyl gallate isomer 2 ^c^	9.4	0.9 ± 0.3	0.6 ± 0.2	0.7 ± 0.2	NS	183.2 → 124.0
Methylgallic acid sulfate isomer 1 ^c^	2.3	1.9 ± 0.2	1.9 ± 0.2	1.2 ± 0.2	<0.05	263.1 → 168.0
Methylgallic acid sulfate isomer 2 ^c^	4.8	2.0 ± 0.6	2.7 ± 0.5	23.2 ± 11.4	NS	263.1 → 168.0
Methylgallic acid sulfate isomer 3 ^c^	5.2	1.4 ± 0.6	0.6 ± 0.1	1.8 ± 0.6	<0.05	263.1 → 168.0
Methylgallic acid sulfate isomer 4 ^c^	6.8	0.6 ± 0.1	0.9 ± 0.2	0.5 ± 0.2	NS	263.1 → 168.0
Methylgallic acid sulfate isomer 5 ^c^	7.7	3.8 ± 0.7	4.6 ± 0.9	4.3 ± 1.4	NS	263.1 → 168.0
Syringic acid (3,5-Dimethoxy-4-hydroxybenzoic acid) ^m^	2.4	3.6 ± 0.4	5.9 ± 0.8	1.7 ± 0.2	<0.05	197.0 → 137.0
**Total**		**299.3 ± 40.6**	**181.2 ± 25.7**	**253.2 ± 47.5**	**NS**	
**Phenylacetic acid Derivatives**						
* 3,4-Dihydroxyphenylacetic acid ^d^	4.2	8.0 ± 1.6	4.5 ± 0.8	3.8 ± 0.4	NS	167.0 → 123.0
* Dihydroxyphenylacetic acid isomer 1 ^d^	1.4	4.7 ± 0.4	5.1 ± 0.4	2.6 ± 0.1	<0.05	167.0 → 123.0
* Dihydroxyphenylacetic acid isomer 2 ^d^	2.9	2.3 ± 0.3	2.0 ± 0.2	1.5 ± 0.2	NS	167.0 → 123.0
Hydroxymethoxyphenylacetic acid sulfate isomer 1 ^b^	1.8	19.5 ± 2.6	14.9 ± 2.3	9.7 ± 1.4	<0.05	261.0 → 181.0
Hydroxymethoxyphenylacetic acid sulfate isomer 2 ^b^	4.5	8.7 ± 2.2	7.6 ± 1.5	8.9 ± 1.8	NS	261.0 → 181.0
Hydroxymethoxyphenylacetic acid sulfate isomer 3 ^b^	5.4	7.8 ± 3.7	2.9 ± 0.8	5.5 ± 1.3	NS	261.0 → 181.0
Hydroxymethoxyphenylacetic acid sulfate isomer 4 ^b^	6.2	20.1 ± 4.2	12.4 ± 2.6	56.7 ± 18.2	<0.05	261.0 → 181.0
Hydroxymethoxyphenylacetic acid sulfate isomer 5 ^b^	7.6	21.2 ± 5.0	11.5 ± 2.9	39.2 ± 10.1	<0.05	261.0 → 181.0
* 4-Hydroxyphenylacetic acid ^e^	6.5	4.2 ± 1.3	2.6 ± 0.6	3.0 ± 0.9	NS	151.0 → 107.0
*Hydroxyphenylacetic acid isomer 1^e^	1.5	45.7 ± 8.1	42.0 ± 6.2	26.1 ± 3.9	NS	151.0 → 108.0
Methylphenylacetic acid sulfate isomer 1 ^e^	3.6	262.2 ± 44.6	166.8 ± 34.0	201.3 ± 37.3	NS	229.0 → 149.0
Methylphenylacetic acid sulfate isomer 2 ^e^	4.4	61.7 ± 9.3	39.6 ± 7.8	50.8 ± 11.3	NS	229.0 → 149.0
Methylphenylacetic acid sulfate isomer 3 ^e^	6.7	19.6 ± 3.8	12.9 ± 2.8	10.2 ± 1.5	NS	229.0 → 149.0
Methylphenylacetic acid sulfate isomer 4 ^e^	10.7	16.1 ± 3.1	14.2 ± 3.4	37.7 ± 11.2	NS	229.0 → 149.0
Methylphenylacetic acid sulfate isomer 5 ^e^	13.6	141.4 ± 71.2	74.0 ± 24.0	265.4 ± 126.5	NS	229.0 → 149.0
Methylphenylacetic acid sulfate isomer 6 ^e^	14.5	13.8 ± 4	6.8 ± 2.5	16.5 ± 3.9	<0.05	229.0 → 149.0
Methylphenylacetic acid glucuronide isomer 1 ^e^	1.9	14.8 ± 9	10.6 ± 2.1	3.5 ± 0.9	<0.05	325.1 → 149.0
Methylphenylacetic acid glucuronide isomer 2 ^e^	7.3	1.6 ± 3.0	2.0 ± 0.8	2.6 ± 0.8	NS	325.1 → 149.0
Methylphenylacetic acid glucuronide isomer 3 ^e^	9.7	4.1 ± 1.2	2.2 ± 0.6	3.6 ± 1.3	NS	325.1 → 149.0
Methylphenylacetic acid glucuronide isomer 4 ^e^	10.7	3.3 ± 1.5	1.7 ± 0.8	5.0 ± 2.5	NS	325.1 → 149.0
Methylphenylacetic acid glucuronide isomer 5 ^e^	11.2	1.0 ± 0.3	0.4 ± 0.1	2.3 ± 0.9	<0.05	325.1 → 149.0
Methylphenylacetic acid glucuronide isomer 6 ^e^	14.7	20.3 ± 7.5	25.4 ± 17.8	24.2 ± 11.0	NS	325.1 → 149.0
**Total**		**702.1 ± 120.0**	**462.1 ± 67.5**	**780.1 ± 158.8**	**NS**	
**Phenylpropanoic acid Derivatives**						
* 3-Hydroxyphenylpropanoic acid ^f^	8.0	3.1 ± 0.8	2.0 ± 0.3	4.4 ± 1.9	NS	165.1 → 121.0
* Hydroxyphenylpropanoic acid isomer 1 ^f^	5.5	4.0 ± 0.6	6.3 ± 2.4	8.2 ± 5.8	NS	165.1 → 121.0
* Hydroxyphenylpropanoic acid isomer 2 ^f^	6.5	9.7 ± 2.0	12.3 ± 4.6	40.5 ± 26.9	NS	165.1 → 121.0
* Hydroxyphenylpropanoic acid isomer 3 ^f^	6.9	1.7 ± 0.9	2.8 ± 1.8	1.3 ± 0.4	NS	165.1 → 121.0
Hydroxyphenylpropanoic acid sulfate isomer 1 ^f^	2.4	55.5 ± 9.5	33.0 ± 8.4	18.7 ± 2.7	NS	245.0 → 165.0
Hydroxyphenylpropanoic acid sulfate isomer 2 ^f^	9.0	57.9 ± 21.5	44.9 ± 18.8	72.2 ± 33.2	NS	245.0 → 165.0
Hydroxyphenylpropanoic acid sulfate isomer 3 ^f^	10.3	42.1 ± 18.3	30.4 ± 12.0	39.3 ± 15.1	NS	245.0 → 165.0
Hydroxyphenylpropanoic acid sulfate isomer 4 ^f^	13.8	57.9 ± 14.1	106.3 ± 23.7	68.0 ± 21.7	NS	245.0 → 165.0
Hydroxyphenylpropanoic acid glucuronide isomer 1 ^m^	3.3	0.5 ± 0.1	0.3 ± 0.1	0.3 ± 0.1	NS	357.1 → 113.0
Hydroxyphenylpropanoic acid glucuronide isomer 2 ^m^	4.8	0.4 ± 0.1	0.1 ± 0.0	0.3 ± 0.0	<0.05	357.1 → 113.0
Hydroxyphenylpropanoic acid glucuronide isomer 3 ^m^	4.3	0.1 ± 0.0	0.1 ± 0.0	0.1 ± 0.0	<0.05	357.1 → 113.0
Hydroxyphenylpropanoic acid glucuronide isomer 4 ^m^	5.8	0.2 ± 0.1	0.1 ± 0.0	0.2 ± 0.1	<0.05	357.1 → 113.0
Hydroxyphenylpropanoic acid glucuronide isomer 5 ^m^	6.6	0.1 ± 0.1	0.04 ± 0.01	0.2 ± 0.1	<0.05	357.1 → 113.0
Dihydroxyphenylpropanoic acid isomer 1 ^m^	3.1	0.1 ± 0.0	0.1 ± 0.0	0.1 ± 0.0	NS	181.1 → 119.0
Dihydroxyphenylpropanoic acid isomer 2 ^m^	4.0	4.4 ± 1.3	1.5 ± 0.3	3.6 ± 0.8	<0.05	181.1 → 119.0
Dihydroxyphenylpropanoic acid isomer 3 ^m^	4.8	0.1 ± 0.1	0.04 ± 0.02	0.1 ± 0.1	NS	181.1 → 119.0
Hydroxymethoxyphenylpropanoic acid isomer 1 ^n^	3.8	0.9 ± 0.1	0.5 ± 0.1	1.0 ± 0.3	NS	195.1 → 121.0
Hydroxymethoxyphenylpropanoic acid isomer 2 ^n^	8.2	0.8 ± 0.2	0.8 ± 0.2	0.8 ± 0.2	NS	195.1 → 121.0
Hydroxymethoxyphenylpropanoic acid isomer 3 ^n^	10.5	0.6 ± 0.2	1.1 ± 0.6	1.3 ± 0.8	NS	195.1 → 121.0
Methoxyphenylpropanoic acid glucuronide isomer 1 ^f^	4.4	2.5 ± 0.8	1.6 ± 0.3	2.1 ± 1.2	NS	371.1 → 195.0
Methoxyphenylpropanoic acid glucuronide isomer 2 ^f^	5.4	4.9 ± 1.1	3.8 ± 0.9	5.3 ± 1.8	NS	371.1 → 195.0
Methoxyphenylpropanoic acid glucuronide isomer 3 ^f^	6.5	8.4 ± 1.9	6.0 ± 1.4	22.4 ± 14.9	NS	371.1 → 195.0
Methoxyphenylpropanoic acid sulfate isomer 1 ^f^	4.9	10.8 ± 2.5	9.5 ± 2.1	13.3 ± 5.3	NS	275.0 → 195.0
Methoxyphenylpropanoic acid sulfate isomer 2 ^f^	6.2	2.7 ± 1.3	0.8 ± 0.4	Not detected	N/A	275.0 → 195.0
Methoxyphenylpropanoic acid sulfate isomer 3 ^f^	6.9	39.3 ± 15.3	23.2 ± 5.6	49.8 ± 13.8	NS	275.0 → 195.0
Methoxyphenylpropanoic acid sulfate isomer 4 ^f^	8.4	14.2 ± 8.4	16.6 ± 10.1	6.5 ± 2.0	NS	275.0 → 195.0
**Total**		**322.7 ± 44.7**	**304.0 ± 50.2**	**360.0 ± 79.3**	**NS**	
**Benzaldehyde Derivatives**						
* 4-Hydroxybenzaldehyde ^g^	6.6	0.2 ± 0.1	0.2 ± 0.0	0.2 ± 0.0	NS	121.0 → 92.0
**Total**		**0.2 ± 0.1**	**0.2 ± 0.1**	**0.3 ± 0.0**	**NS**	
**Pyrogallol Derivatives**						
Pyrogallol sulfate isomer 1 ^h^	1.7	107.1 ± 16.5	84.5 ± 15.9	84.1 ± 18.8	NS	205. 0 → 125.0
Pyrogallol sulfate isomer 2 ^h^	2.6	93.2 ± 30.5	105.8 ± 19.1	80.9 ± 30.4	NS	205. 0 → 125.0
Pyrogallol sulfate isomer 3 ^h^	4.9	3377.4 ± 1910.4	4728.0 ± 1052.2	8496.0 ± 2091.8	<0.05	205. 0 → 125.0
Pyrogallol sulfate isomer 4 ^h^	7.7	21.5 ± 5.2	10.5 ± 2.1	16.6 ± 2.9	NS	205. 0 → 125.0
Methylpyrogallol sulfate isomer 1 ^h^	3.2	138.4 ± 23.8	130.6 ± 27.3	83.1 ± 33.0	NS	219.2 → 139.0
Methylpyrogallol sulfate isomer 2 ^h^	3.9	119.3 ± 60.6	82.3 ± 20.0	134.7 ± 31.7	NS	219.2 → 139.0
Methylpyrogallol sulfate isomer 3 ^h^	5.8	279.4 ± 152.6	421.8 ± 104.3	520.8 ± 98.9	NS	219.2 → 139.0
Methylpyrogallol sulfate isomer 4 ^h^	8.2	22.6 ± 6.9	8.1 ± 2.4	30.3 ± 10.6	NS	219.2 → 139.0
Methylpyrogallol glucuronide isomer 1 ^h^	5.0	Not detected	1.7 ± 1.0	9.5 ± 1.5	<0.05	315.1 → 216.9
Methylpyrogallol glucuronide isomer 2 ^h^	5.4	Not detected	0.6 ± 0.6	0.9 ± 0.6	NS	315.1 → 216.9
**Total**		**4158.9 ± 2151.9**	**5574.0 ± 1196.6**	**9456.9 ± 2208.5**	**<0.05**	
**Catechol Derivatives**						
* Catechol (Benzene-1,2-diol) ^h^	4.1	18.7 ± 12.2	15.7 ± 4.1	15.1 ± 4.0	NS	109.0 → 108.0
Catechol methyl sulfate isomer 1 ^h^	5.7	1189.1 ± 1011.8	694.8 ± 244.2	768.9 ± 252.8	NS	203.0 → 123.0
Catechol methyl sulfate isomer 2 ^h^	7.7	8228. 2 ± 1836.0	4126.2 ± 827.0	5717. 8 ± 903.8	NS	203.0 → 123.0
Catechol sulfate isomer 1 ^h^	2.6	442.0 ± 118.7	265.0 ± 77.6	337.5 ± 76.5	NS	189.0 → 109.0
Catechol sulfate isomer 2 ^h^	3.5	36.4 ± 21.6	93.8 ± 58.3	664.8 ± 575.0	NS	189.0 → 109.0
Catechol sulfate isomer 3 ^h^	4.6	42919.4 ± 26,920.7	26,826.7 ± 5601.6	35,686.6 ± 8264.9	NS	189.0 → 109.0
Catechol sulfate isomer 4 ^h^	8.5	287.3 ± 79.8	182.0 ± 50.9	201.2 ± 44.3	NS	189.0 → 109.0
Catechol glucuronide isomer 1 ^h^	1.7	38.7 ± 5.8	36.0 ± 5.8	14.7 ± 3.8	<0.05	285.1 → 109.0
Catechol glucuronide isomer 2 ^h^	2.4	0.1 ± 0.1	2.2 ± 0.9	2.8 ± 2.3	NS	285.1 → 109.0
Catechol glucuronide isomer 3 ^h^	4.3	123.2 ± 116.6	14.6 ± 6.8	15.7 ± 5.7	NS	285.1 → 109.0
Catechol glucuronide isomer 4 ^h^	6.7	1.4 ± 0.6	0.8 ± 0.4	1.4 ± 0.4	NS	285.1 → 109.0
Dicatechol sulfate isomer 1 ^h^	3.8	21.5 ± 4.3	14.2 ± 3.0	14.8 ± 4.2	NS	299.0 → 109.0
Dicatechol sulfate isomer 2 ^h^	4.3	5.2 ± 1.4	1.1 ± 0.5	2.3 ± 0.8	NS	299.0 → 109.0
Dicatechol sulfate isomer 3 ^h^	5.2	15.9 ± 2.1	5.1 ± 1.0	3.1 ± 0.5	<0.05	299.0 → 109.0
**Total**		**53327.3 ± 28063.0**	**32278.3 ± 5964.0**	**43446.6 ± 9154.8**	**NS**	
**Hippuric acid Derivatives**						
* Hippuric acid ^i^	5.1	2805. 9 ± 568.8	3706.9 ± 582.3	2023.6 ± 241.9	<0.05	178.1 → 77.0
Hippuric acid conjugate 1 ^i^	1.3	1.3 ± 0.4	0.8 ± 0.4	0.4 ± 0.1	NS	311.0 → 135.0
Hippuric acid conjugate 2 ^i^	4.6	0.2 ± 0.1	11.7 ± 11.2	1.6 ± 1.2	NS	311.0 → 135.0
Hippuric acid conjugate 3 ^i^	8.8	0.3 ± 0.3	Not detected	0.4 ± 0.2	N/A	311.0 → 135.0
Hippuric acid conjugate 4 ^i^	13.6	Not detected	Not detected	0.4 ± 0.3	N/A	311.0 → 135.0
Hippuric acid sulfate isomer 1 ^i^	2.1	6.9 ± 2.9	1.9 ± 0.8	4.9 ± 2.8	NS	258.0 → 178.0
Hippuric acid sulfate isomer 2 ^i^	3.5	135.0 ± 100.0	71.0 ± 47.5	53.6 ± 26.3	NS	258.0 → 178.0
Hippuric acid sulfate isomer 3 ^i^	5.2	59.5 ± 22.1	39.9 ± 13.5	20.4 ± 6.9	NS	258.0 → 178.0
Hippuric acid sulfate isomer 4 ^i^	6.3	31.5 ± 16.8	13.7 ± 7.7	15.7 ± 5.4	NS	258.0 → 178.0
Hippuric acid sulfate isomer 5 ^i^	7.3	Not detected	1.7 ± 1.2	26.1 ± 6.4	<0.05	258.0 → 178.0
Hippuric acid sulfate isomer 6 ^i^	7.2	126.3 ± 71.9	36.2 ± 14.9	25.7 ± 6.4	NS	258.0 → 178.0
Hydroxyhippuric acid isomer 1 ^i^	3.6	180.0 ± 63.2	120.3 ± 34.4	193.4 ± 36.4	NS	194.0 → 93.0
Hydroxyhippuric acid isomer 2 ^i^	4.0	122.3 ± 42.2	40.6 ± 11.2	159.3 ± 31.1	<0.05	194.0 → 93.0
Hydroxyhippuric acid isomer 3 ^i^	8.1	377.3 ± 180.7	262.6 ± 62.5	1.2 ± 160/7	NS	194.0 → 93.0
* 4-Methylhippuric acid ^t^	7.9	2.0 ± 1.0	1.5 ± 0.9	1.2 ± 0.4	NS	192.0 → 91.0
Methyl hippurate ^i^	6.6	2.8 ± 2.3	7.8 ± 2.1	6.3 ± 1.7	NS	192.1 → 77.0
**Total**		**3851.3 ± 681.7**	**4316.8 ± 625.7**	**2951.4 ± 306.3**	**<0.05**	
**Cinnamic acid Derivatives**						
* Caffeic acid (3’,4’-Dihydroxycinnamic acid) ^m^	6.6	0.1 ± 0.0	Not detected	0.1 ± 0.0	N/A	179.0 → 135.0
* Caffeic acid isomer ^m^	4.9	1.0 ± 0.2	1.4 ± 0.2	0.6 ± 0.1	<0.05	179.0 → 135.0
* Ferulic acid (4’-Hydroxy-3’-methoxycinnamic acid) ^n^	10.2	0.5 ± 0.1	0.4 ± 0.1	0.4 ± 0.1	NS	193.1 → 134.0
* Ferulic acid isomer 1 ^n^	8.4	1.4 ± 0.4	0.9 ± 0.1	2.5 ± 1.0	NS	193.1 → 134.0
* Ferulic acid isomer 2 ^n^	12.2	0.5 ± 0.1	0.5 ± 0.1	0.2 ± 0.0	<0.05	193.1 → 134.0
Methoxycinnamic acid glucuronide isomer 1 ^n^	5.6	4.5 ± 1.7	3.7 ± 0.9	7.6 ± 3.4	NS	369.1 → 193.0
Methoxycinnamic acid glucuronide isomer 2 ^n^	6.9	3.3 ± 1.1	2.4 ± 0.4	5.5 ± 3.0	NS	369.1 → 193.0
Methoxycinnamic acid glucuronide isomer 3 ^n^	7.5	0.4 ± 0.1	0.5 ± 0.1	0.3 ± 0.1	NS	369.1 → 193.0
Methoxycinnamic acid glucuronide isomer 4 ^n^	8.6	1.0 ± 0.3	0.4 ± 0.2	0.7 ± 0.3	NS	369.1 → 193.0
Methoxycinnamic acid glucuronide isomer 5 ^n^	9.6	0.7 ± 0.2	0.5 ± 0.1	1.0 ± 0.4	NS	369.1 → 193.0
Methoxycinnamic acid sulfate isomer 1 ^n^	11.0	2.9 ± 1.4	1.0 ± 0.3	9.3 ± 7.3	NS	273.0 → 193.0
Methoxycinnamic acid sulfate isomer 2 ^n^	11.9	3.7 ± 0.9	1.2 ± 0.4	3.6 ± 1.5	NS	273.0 → 193.0
Ferulic acid hexoside isomer 1 ^n^	4.7	0.7 ± 0.1	0.6 ± 0.1	0.6 ± 0.1	NS	355.1 → 135.0
Ferulic acid hexoside isomer 2 ^n^	5.8	1.4 ± 0.3	1.9 ± 0.7	5.2 ± 2.9	NS	355.1 → 135.0
Ferulic acid hexoside isomer 3 ^n^	6.1	0.6 ± 0.3	1.1 ± 0.6	2.2 ± 1.4	NS	355.1 → 135.0
Ferulic acid hexoside isomer 4 ^n^	8.5	0.4 ± 0.1	0.3 ± 0.1	0.3 ± 0.1	NS	355.1 → 135.0
Ferulic acid hexoside isomer 5 ^n^	12.5	0.9 ± 0.2	0.8 ± 0.3	1.5 ± 0.8	NS	355.1 → 135.0
Ferulic acid derivative isomer 1 ^n^	4.0	11.7 ± 2.6	7.1 ± 1.7	9.9 ± 2.5	NS	211.1 → 136.0
Ferulic acid derivative isomer 2 ^n^	4.6	6.2 ± 2.3	2.0 ± 0.4	17.3 ± 14.3	NS	211.1 → 136.0
* *p*-Coumaric acid (4’-Hydroxycinnamic acid) ^j^	8.8	0.5 ± 0.1	0.4 ± 0.1	0.3 ± 0.0	<0.05	163.0 → 119.0
* *m*-Coumaric acid (3’-Hydroxycinnamic acid) ^k^	10.0	0.8 ± 0.1	0.7 ± 0.1	0.7 ± 0.1	NS	163.0 → 119.0
* *o*-Coumaric acid (2’-Hydroxycinnamic acid) ^l^	11.5	0.9 ± 0.1	0.9 ± 0.0	0.5 ± 0.1	<0.05	163.0 → 119.0
* Coumaric acid isomer 1 ^j^	8.2	3.4 ± 2.7	1.4 ± 0.8	0.6 ± 0.1	NS	163.0 → 119.0
**Total**		**47.3 ± 9.2**	**30.2 ± 3.3**	**70.9 ± 27.8**	**NS**	
**Valerolactone Derivatives**						
Trihydroxyphenyl-*γ*-valerolactone sulfate isomer 1 ^p^	5.7	Not detected	Not detected	0.3 ± 0.2	N/A	303.0 → 223.1
Trihydroxyphenyl-*γ*-valerolactone sulfate isomer 2 ^p^	12.0	0.05 ± 0.04	0.03 ± 0.02	0.03 ± 0.01	NS	303.0 → 223.1
Dihydroxyphenyl-*γ*-valerolactone glucuronide isomer 1 ^p^	4.5	Not detected	Not detected	0.1 ± 0.1	N/A	383.1 → 207.1
Dihydroxyphenyl-*γ*-valerolactone glucuronide isomer 2 ^p^	5.9	0.1 ± 0.1	0.01 ± 0.01	0.2 ± 0.1	NS	383.1 → 207.1
Dihydroxyphenyl-*γ*-valerolactone glucuronide isomer 3 ^p^	6.2	0.6 ± 0.3	0.02 ± 0.01	1.3 ± 0.5	<0.05	383.1 → 207.1
Dihydroxyphenyl-*γ*-valerolactone sulfate isomer 1 ^o^	6.3	1.1 ± 0.6	0.7 ± 0.7	3.8 ± 2.6	NS	287.0 → 207.0
Dihydroxyphenyl-*γ*-valerolactone sulfate isomer 2 ^o^	7.8	62.3 ± 20.5	13.3 ± 4.5	68.7 ± 21.8	<0.05	287.0 → 207.0
**Total**		**64.1 ± 20.7**	**14.1 ± 4.7**	**74.4 ± 24.1**	**<0.05**	
**Others**						
Abscisic acid hexoside ^p^	13.0	0.7 ± 0.2	0.5 ± 0.1	0.3 ± 0.1	NS	425.2 → 219.0
Abscisic acid sulfate isomer 1 ^p^	1.6	13.4 ± 3.4	9.4 ± 1.3	8.2 ± 1.3	NS	255.0 → 115.0
Abscisic acid sulfate isomer 2 ^p^	1.9	Not detected	Not detected	10.7 ± 2.8	N/A	255.0 → 115.0
**Total**		**14.1 ± 3.5**	**9.9 ± 1.4**	**19.1 ± 2.8**	**<0.05**	
**Total for all compounds**		**62,787.4 ± 31135.4**	**43,170.7 ± 7939.3**	**57,412.7 ± 12010.0**	**NS**	

Compounds for which commercial standards were not available were quantified using the standards sharing similar chemical structure and molecular weight: ^a^ 4-hydroxybenzoic acid, ^b^ vanillic acid (4-hydroxy-3-methoxybenzoic acid), ^c^ gallic acid (3,4,5-trihydroxybenzoic acid), ^d^ 3,4-dihydroxyphenylacetic acid, ^e^ 4-hydroxyphenylacetic acid, ^f^ 3-hydroxyphenylpropanoic acid, ^g^ 4-hydroxybenzaldehyde, ^h^ catechol (benzene-1,2-diol), ^i^ hippuric acid, ^j^ *p*-coumaric acid (4’-hydroxycinnamic acid), ^k^ *m*-coumaric acid (3’-hydroxycinnamic acid), ^l^ *o*-coumaric acid (2’-hydroxycinnamic acid), ^m^ caffeic acid (3’,4’-dihydroxycinnamic acid), ^n^ ferulic acid (4’-hydroxy-3’-methoxycinnamic acid), ^o^ catechin, ^p^ chlorogenic acid, ^q^ 3,4-dihydroxybenzoic acid, ^r^ 2,5-dihydroxybenzoic acid, ^s^ 2,3-dihydroxybenzoic acid, ^t^ 4-methylhippuric acid. All the compounds in this table were quantified in negative ion mode. * Quantified using corresponding standards or share the same transition of the standards. NS: not significant, N/A: not applicable.

**Table A3:** Dietary compositions of study test beverages, breakfast and lunch.

Item Name	Quantity	Measure	Wgt (g)	Cals (kcal)	FatCals (kcal)	Prot (g)	Carb (g)	TotFib (g)	Sugar (g)	SugAdd (g)	Fat (g)
Test beverage—MP			600.0	321.4	0	0	85.7	7.1	75.0	0	0
Water, tap	100	Gram	100.0	0	0	0	0	0	0	0	0
Mango, frozen	500	Gram	500.0	321.4	0	0	85.7	7.1	75.0	--	0
Test beverage—MP + VC			600.1	321.4	0	0	85.7	7.1	75.0	0	0
Mango, frozen	500	Gram	500.0	321.4	0	0	85.7	7.1	75.0	--	0
Ascorbic acid, VC, food grade	100	Milligram	0.1	0	0	0	0	0	0	--	0
Water, tap	100	Gram	100.0	0	0	0	0	0	0	0	0
Test beverage—VC			600.1	286.9	0	0	75.0	0	73.1	61.6	0
Sugar, white, granulated	38	Gram	38.0	147.1	0	0	38.0	0	37.9	37.9	0
Fructose, powder	25.5	Gram	25.5	93.8	0	0	25.5	0	23.6	23.6	0
Dextrose	11.5	Gram	11.5	46	--	0	11.5	0	11.5	--	--
Ascorbic acid, VC, food grade	100	Milligram	0.1	0	0	0	0	0	0	--	0
Water, tap	525	Gram	525.0	0	0	0	0	0	0	0	0
Breakfast			247.0	568.2	216.1	16.7	74.6	0	32.1	0	24.0
Biscuits, buttermilk, frozen	2	Each	118.0	340.0	144.0	8.0	44.0	--	4.0	--	16.0
Butter, unsalted, sweet cream	8	Gram	8.0	57.1	56.6	0	--	0	0	--	6.3
Jelly, apple	46	Gram	46.0	115.0	0	0	29.9	0	27.6	--	0
Egg white, raw	70	Gram	70.0	36.4	1.1	7.6	0.5	0	0.5	0	0.1
Cheese, cheddar, sharp, finely shredded	5	Gram	5.0	19.6	14.5	1.1	0.2	0	0	0	1.6
Lunch			243.0	244.0	173.9	8.2	10.3	3.4	4.3	0	19.3
Cucumber, fresh, without skin, sliced	200	Gram	200.0	24.0	2.9	1.2	4.3	1.4	2.8	0	0.3
Dressing, cucumber ranch, Hidden valley	15	Gram	15.0	50.0	45.0	0	1.0	--	0.5	--	5.0
Peanuts, cocktail, lightly salted	28	Gram	28.0	170.0	126.0	7.0	5.0	2.0	1.0	--	14.0

Wgt—weight; Cals—calories; FatCals—calories from fat; Prot—protein; Carb—carbohydrates; TotFib—total fiber; SugAdd—sugar added; MP—mango pulp; VC—vitamin C.

**Table A4:** Standards used for the quantification of (poly)phenolic metabolites with their limits of quantification (LOQ).

Standard	LOQ (nmol/L)	Compounds Quantified
2,3-Dihydroxybenzoic acid	20.3	2,3-Dihydroxybenzoic acid
2,5-Dihydroxybenzoic acid	81.2	2,5-Dihydroxybenzoic acid
4-Methylhippuric acid	64.8	4-Methylhippuric acid, dihydroxybenzoic acid sulfate isomers
3,4-Dihydroxybenzoic acid	20.3	3,4-Dihydroxybenzoic acid
3,4-Dihydroxyphenylacetic acid	74.4	3,4-Dihydroxyphenylacetic acid, dihydroxyphenylacetic acid isomers, methoxybenzoic acid glucuronide isomers, methoxybenzoic acid sulfate isomers
3-Hydroxyphenylpropanoic acid	602.0	3-Hydroxyphenylpropanoic acid, hydroxyphenylpropanoic acid isomers, methoxyphenylpropanoic acid glucuronide isomers, methoxyphenylpropanoic acid sulfate isomers, hydroxyphenylpropanoic acid sulfate isomers
4-Hydroxybenzaldehyde	102.5	4-Hydroxybenzaldehyde
4-Hydroxyphenylacetic acid	10.3	4-Hydroxyphenylacetic acid, hydroxyphenylacetic acid isomer, methylphenylacetic acid sulfate isomers, methylphenylacetic acid glucuronide isomers
Caffeic acid	69.4	Caffeic acid isomers, dihydroxyphenylpropanoic acid isomers, hydroxyphenylpropanoic glucuronide isomers, syringic acid
Catechin	86.2	Dihydroxyphenyl-*γ*-valerolactone sulfate isomers
Catechol	14.2	Catechol, catechol methyl sulfate isomers, catechol sulfate isomers, dicatechol sulfate isomers, catechol glucuronide isomers, pyrogallol sulfate isomers, methylpyrogallol sulfate isomers, methylpyrogallol glucuronide isomers
Chlorogenic acid	4.4	Trihydroxyphenyl-*r*-valerolactone sulfate isomers, dihydroxyphenyl-*γ*-valerolactone glucuronide isomers, dihydroxyphenyl-*r*-valerolactone sulfate isomers, abscisic acid hexoside, abscisic acid sulfate isomers
Ferulic acid	8.0	Ferulic acid isomers, methoxycinnamic acid glucuronide isomers, ferulic acid hexoside isomers, ferulic acid derivative isomers, methoxycinnamic acid sulfate isomers, hydroxymethoxyphenylpropanoic acid isomers
Gallic acid	36.8	Gallic acid, gallic acid derivative isomers, galloylshikimic acid isomers, galloyl glucose isomers, methyl gallate isomers, methylgallic acid sulfate isomers
Hippuric acid	34.9	Hippuric acid, hippuric acid conjugates, methyl hippurate, hippuric acid sulfate isomers, hydroxyhippuric acid isomers
4-Hydroxybenzoic acid	90.6	4-Hydroxybenzoic acid, hydroxybenzoic acid isomers, hydroxybenzoic acid hexoside, hydroxybenzoic acid sulfate isomers, benzoic acid glucuronide isomers
Mangiferin	5.2	Mangiferin
*m*-Coumaric acid	38.1	*m*-Coumaric acid
*o*-Coumaric acid	38.1	*o*-Coumaric acid
*p*-Coumaric acid	9.5	*p*-Coumaric acid, coumaric acid isomer
Vanillic acid	148.8	Iso/vanillic acid isomers, hydroxymethoxyphenylacetic acid sulfate isomers
